# Eco-Sanitary Regionalization of Wild Boar (*Sus scrofa*) in the Western Palearctic Realm as a Tool for the Stewardship of African Swine Fever

**DOI:** 10.1155/2023/8080496

**Published:** 2023-03-22

**Authors:** Cecilia Aguilar-Vega, Carolina Muñoz-Pérez, José Manuel Sánchez-Vizcaíno, Marta Martínez-Avilés, Jaime Bosch

**Affiliations:** ^1^VISAVET Health Surveillance Centre and Animal Health Department, Complutense University of Madrid, Madrid, Spain; ^2^Animal Health Research Center (CISA-INIA-CSIC), Madrid, Spain

## Abstract

African swine fever (ASF) is a viral hemorrhagic disease affecting domestic and wild suids, with catastrophic socioeconomic impact worldwide. In the European scenario, wild boar (*Sus scrofa*) actively contributes to the maintenance and spread of the disease. In this study, we applied a multivariate clustering method to define ecoregions in the western Palearctic realm based on ecological and sanitary aspects of ASF in wild boar. Beforehand, we performed a principal components analysis for the eight selected variables. An analysis of the countries was carried out in terms of the extent of ecoregions and ASF notifications. After clustering, we identified nine eco-regions that showed important differences based on the used eco-sanitary variables. Several ecoregions stand out in the country and notification analysis for retaining the most ASF cases and being present in their surrounding locations. Here, we developed a cartographical tool to understand patterns in the distribution and spread of ASF in wild boar at the European level, as well as improve prevention, control, surveillance plans, and eradication strategies, especially future vaccination programs once a safe and effective vaccine is available.

## 1. Introduction

African swine fever (ASF) is a viral hemorrhagic disease affecting domestic pigs and wild boars. Its etiological agent is the African swine fever virus (ASFV), a double-stranded DNA virus that belongs to the Asfarviridae family [1]. Due to the high lethality of some strains, the lack of an effective vaccine and treatment, and the severe economic consequences, it is a notifiable disease to the World Organization for Animal Health (WOAH funded as OIE). Since its introduction in the Caucasus in 2007 [2], the continuing spread of the disease has been unstoppable, reaching a pandemic dimension. ASF is currently present on five continents [3], posing a major threat to the global swine industry and highlighting the need for new epidemiological tools to control and eradicate the disease [4].

Wild populations of suids are key in many regions for the maintenance and spread of the disease. In Europe, wild boars are a wildlife reservoir of ASF that has spread in some regions [5]. This implies a continued risk of spillover into domestic pig holdings, particularly into open swine production systems or with low biosecurity levels [6]. However, it should be noted that the wild-domestic interface can be bi-directional and pathogen transmission can exist from domestic pigs to wild boars too [7]. Although limited information is available about wild boar ASF notifications in Asia, studies have confirmed the presence of ASFV in Asian wild boar populations [8, 9].

The development of certain epidemiological tools for wild pigs can help with the implementation of control measures in the field, such as oral mass vaccination programs for free-ranging wild boars. These vaccination programs could be a promising strategy to control ASF worldwide, as was the case for classical swine fever control during the 2000s [10]. On that note, eco-sanitary regions are defined as areas with similar biotic and abiotic feature interactions, such as climate, orography, topography, geology, or vegetation [11]. Until now, ecoregions have been commonly used as a tool for conservation [12], but they can also have shown to be applicable to the management of veterinary and ecological diseases, particularly those which have been linked to climatic factors like vector-borne diseases or diseases with wild reservoirs [13]. ASF in wild boar habitats depends to a large extent on environmental factors such as wild reservoir presence and the capacity of virus survival in the environment [14], which are both extremely linked to climate and the environment at the same time. The final aim of this study was to obtain and identify the eco-regionalization in the Western Palearctic realm according to variables related to ecological and sanitary aspects of ASF distribution in wild boar to focus the control strategies and surveillance of ASF. Moreover, this study can also be useful to identify potential areas where vaccination strategies for wild boar would be more effective.

## 2. Materials and Methods

### 2.1. Study Area

The area of study focused on the Western Palearctic realm with an extension of 34–72°N, 15.95°W, and 47°E. This is a wide and diverse area in terms of climatic, vegetative, and orographic features. Among the most prevalent climates in the region according to the Köppen–Geiger classification system are subarctic, humid continental, oceanic, Mediterranean, and desert climates [15].

The study area corresponds to agricultural and natural landscapes with high-quality habitats for wild boar in terms of food and shelter. This leads to a greater wild boar abundance in the study area [16] except northern Europe, which has a subarctic climate and lower densities of wild boar. These areas are characterized by deep snow and severe winters that that are difficult to access food resources, which is one of the main causes of mortality in wild boar populations [17].

### 2.2. Variable Selection

We selected eight variables related to ecological and sanitary aspects of ASF distribution in wild boar. We obtained the mean annual temperature from the WorldClim dataset at 1 km^2^ [18]. Temperature facilitates food supply and reduces juvenile mortality, having a positive influence on the presence of wild boar [19]. On the other hand, temperature is one important factor related to the persistence of ASF-positive carcasses in the environment. Carcasses can remain infectious for several months, particularly at low temperatures [20].

The vegetation structure as a percentage of coverage of trees, herbaceous vegetation, and bare terrain at 1 km^2^ spatial resolution was calculated from the Vegetation Continuous Fields [21], using the correspondent categories. Wild boar distribution and behavior are strongly correlated with land cover. Natural, agro-forest, and agricultural landscapes provide wild boars with food and cover [16]. The elevation of the terrain (henceforth altitude) was also considered a relevant ecological variable for the model and retrieved from the Global Land Cover Facility [22] at 1 km^2^ spatial resolution. Altitude is highly correlated with vegetation, temperature, and, in some cases, wild boar presence.

The normalized difference vegetation index (NDVI) is a remote sensing index that indicates biomass vegetation and phenology as well as soil moisture [23]. It was retrieved from the MODIS product dataset [24], the Global Inventory Monitoring and Modeling Studies (GIMMS), using the R package “MODIStsp” [25]. The Quality of Available Habitat (QAH) for wild boar is a cartographic tool designed to improve the species distribution based on the ecology and behavior of the species. We applied here the categories of QAH described in previous work [16], combined with the NDVI to avoid the use of categorical variables in the posterior principal component analysis. Moreover, we applied NDVI with QAH to add temporal and spatial trends and variations in vegetation distribution, productivity, dynamics, degradation, fragmentation, and the ecological effects on the population dynamics of animals. Thus, we obtained a better measure of the impact of humans on the environment and in the habitat of wild boar and their population dynamics and distributions [26]. The QAH categories were reclassified from zero to 100 prior to the multiplication with the NDVI raster.

Other ASF-related variables were chosen for the analysis, among which is the probability of wild boar presence, obtained at 1 km^2^ spatial resolution [27]. Although the probability of presence does not present a linear correlation with abundance, it can serve as a proxy for the distribution of wild boar in the area of study [28]. Finally, we used the mean solar radiation that was calculated using an elevation model [22] and its derivatives of elevation such as slope, orientation, and latitude and longitude maps. Solar radiation variables were generated with the Geographic Resources Analysis Support System [29] software v6.4.1 and implemented in the module “r.sun” [30]; at 1 km^2^ resolution. The decay of carcasses would be negatively correlated with the risk of transmission, and it is heavily influenced by abiotic conditions that play a major role in the degradation of the virus, such as solar radiation: the carcasses exposed to the sun decomposed faster [31, 32].

All rasters were aligned and adjusted to a final resolution of 0.008333° (approximately 1 km^2^) and set to the extension of the area of study prior to any subsequent analysis.

### 2.3. Statistical Analysis

Principal component analysis (PCA) is a technique widely used in the machine learning environment for dimensionality reduction of high dimensional data sets while maintaining most of the initial variability in the data set. This technique transforms a set of correlated variables into a set of uncorrelated ones, named principal components (PCs), which are linear combinations of the original variables [33]. These PCs, or eigenvectors, are linked to eigenvalues, which could be interpreted as the “length” or “magnitude” of these PCs. For the procedure, variable values were first normalized to eliminate data heterogeneity, and all data points of the rasters were used [33]. We selected the number of PCs for further analysis based on the percentage of the retained variance of the original variables by the PCs. After that, the contribution of every original variable to the PCs was obtained.

To define the ecoregions, we used *k*-means clustering, an unsupervised machine learning algorithm. The algorithm identifies groups or clusters of data points (i.e., raster pixels) that contain high intraclass similarity and the highest possible intercluster dissimilarity [34, 35]. *k* needs to be set before performing the algorithm. Thus, the optimal number of clusters (*k*) was selected using the gap statistics method [36], with 10^4^ random data points for the analysis. To define the clusters, we perform the *k*-means algorithm using 10^6^ random data points. Afterward, we characterized each cluster using the original variables.

This process was developed in R version 3.6.3 [37], using the following packages: “raster” [38], “RStoolbox” [39], “cluster” [40], “dplyr” [41], “reshape2” [42]. For visualization purposes, we used the following packages: “ggplot2” [43], “factoextra” [44], “gridExtra” [45], “corrplot” [46], and “RColorBrewer” [47].

### 2.4. Descriptive Analysis of Ecoregions per Country and ASF Notifications

For each country, we estimated the distribution percentage of the different ecoregions by dividing the number of cells in each cluster by the total number of cells per country. This way, we obtained a relative ecoclimatic composition per country. For countries whose extension exceeded the limits of the study (in the eastern and southern borders), only the extent within the study area was included in the calculations.

Records of ASF notifications for wild boar were superimposed on the ecoregion map. We retrieved the geographical coordinates of ASF cases in wild boar from the WAHIS [3], EMPRES-i [48], and ADIS databases [49] from 2007, when ASFV genotype II entered Europe, until May 2022. Since we used databases from different sources, we removed duplicates before the analysis. The predominant transmission mode in wild boar in the EU has been due to wild boar-to-wild boar transmission [5, 16], and we assumed that such transmission could be influenced by the type of ecoclimatic region. We overlapped buffers of 0.045 decimal degree radius (approximately 5 km) around each ASF case in the EU to take into account not only the ecoregion in which they are located but to evaluate the composition in terms of ecoregion of the surrounding area. This can allow a more specific analysis that accounts for potential reporting bias in terms of the geographical accuracy of the ASF cases and excludes from the analysis nonaffected areas within an affected country. We performed a *χ*^2^ goodness-of-fit test to evaluate if there was a difference between the observed ASF cases in wild boar and the expected random cases based on the surface area of each cluster within the buffer. Only sufficiently represented ecoregions were included in this analysis.

In addition, using the ecoregion percentage within the buffers of each country, we estimated the expected percentage of wild boar notifications in each ecoregion based on the surface area of each cluster per country. We then compared the expected probability of cases for each ecoregion with the observed ASF data in wild boar. Of the affected countries, only Greece was excluded from this analysis because of the uncertainty due to the small sample size (only one wild boar case was reported in the time period considered).

## 3. Results

### 3.1. Cluster Definition

Prior to the application of the principal components analysis, we explored the correlation between the eight selected variables (S1 and S2 Figures). The majority of the variables were not strongly correlated (>0.7), except for the mean solar radiation and the mean temperature. We selected the first four principal components for further analysis since they retained 89% of the variance of the original variables.

The contribution of each variable to the principal components is shown in Table 1. PC1 represented treeless areas with high mean solar radiation and mean temperature. PC2 was related to the presence of wild boar, depicting areas with a high-level presence and habitat quality for wild boar and low grassland coverage. PC3 was characterized by grasslands with low altitudes and a low presence of bare areas. PC4 represented warmer regions with low altitudes.

**Figure 1:**
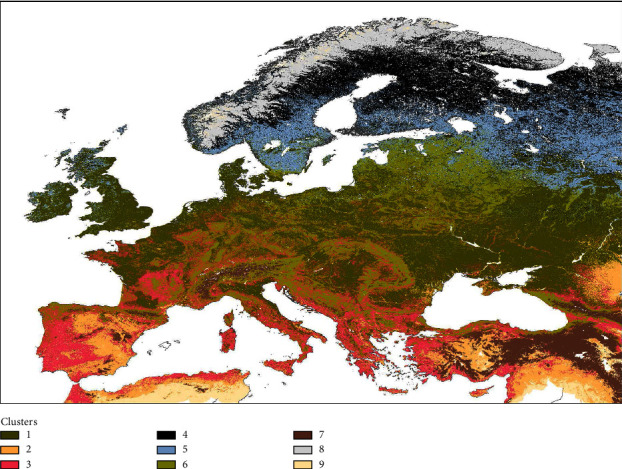
Defined ecoregions with a spatial resolution of 0.0083 degrees. Map generated using ArcMap v10.8.1 (Esri®).

**Figure 2:**
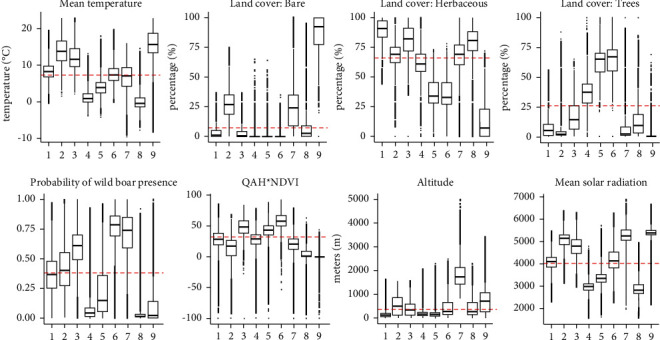
Boxplots representing the values of each ecoregion for the variables included in the analysis. The horizontal dashed line indicates the mean value for each variable.

The optimal number of clusters was nine according to the gap statistics technique (S3 Figure). The nine selected clusters represent different ecoregions based on bioclimatic and ASF-related variables (Figure 1). It can be appreciated the great influence of the different variables in the definition of those ecoregions (Figure 2).

To facilitate the interpretation of this geospatial tool for veterinary services, wildlife management authorities, modelers and decision-makers, these nine ecoregions could be visually grouped into four regions, according to the eco-sanitary geographic areas for wild boar: north Western Palearctic realm (4, 5, 8), central Western Palearctic realm (1, 6), south Western Palearctic realm (2, 3, 9), and the Western Palearctic realm mountainous systems (7) (Figure 1). Areas corresponding to ecoregion 1 are mainly characterized by grasslands with low altitudes and low overall temperatures. Ecoregion 6 represents areas with features that favor the presence of wild boar with high tree coverage. Ecoregion 3 is characterized by warmer regions with grassland and a relatively higher probability of the presence of wild boar and the quality of the habitat for this species. In contrast, areas belonging to ecoregion 7 are characterized by high elevation and high mean solar radiation when compared to the entire study area. Those areas had a relatively high probability of the presence of wild boar alongside lower habitat suitability in comparison. Ecoregion 4 areas are colder with the presence of trees. For this cluster, the probability of the presence of wild boar was low for the majority of point data in contrast with the quality of the habitat which was above the mean for the first two quartiles. The region defined by cluster 5 is less cold than the fourth; however, it is characterized by abundant tree coverage and, in general, a higher QAH. Ecoregion 8 generally corresponds to polar areas where wild boar is not widely distributed. On the other hand, cluster 2 was comprised of areas with a higher percentage of bare soil than other clusters, higher temperatures than the mean and little forest cover in general. The region defined by cluster 9 is characterized by bare areas with a low distribution of wild boar based on the probability of presence and QAH. The majority of data points correspond with warmer areas, although this cluster is also present in high latitudes with colder weather (Figure 1). The description provided here corresponds to the characteristics of most of the data points; however, as presented in Figure 2, there are outliers that can deviate from the general description.

### 3.2. Descriptive Analysis of Ecoregions per Country and ASF Notifications

**Figure 3:**
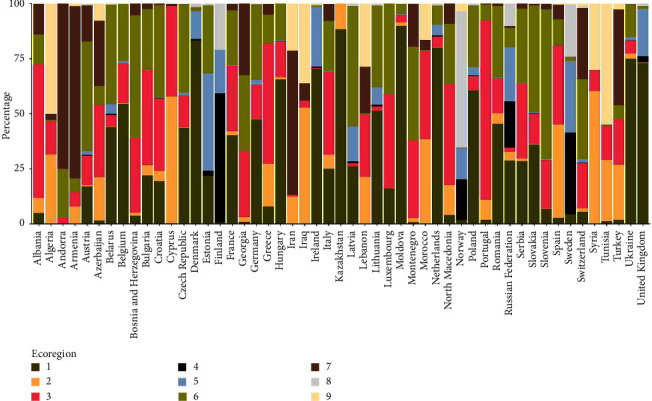
Ecoregion composition of the countries in the study area. Some territories are not shown due to their small surface: Gibraltar, Liechtenstein, Monaco, and Vatican City.

As could be expected from the extension of the study area, there was a high degree of variability in the distribution percentages of the different ecoregions of the countries that were included (Figure 3). However, almost all central Eurasian countries are mainly represented by ecoregion 1, 6, and 3, with the exception of Estonia and Latvia which are also highly represented by ecoregion 5.

For the analysis of ASF wild boar notifications in EU countries, we included 33,995 ASF cases. In the buffer areas more than 98% of the surface corresponded to ecoregions 1, 3, 5, and 6, and we found statistically significant differences in the distribution of cases in those ecoregions in comparison with what would be expected if cases were randomly distributed (Table 2). Ecoregion 6 concentrated remarkably more cases than expected (51.5%), distantly followed by ecoregion 3 (11.43%). In contrast, ecoregions 1 and 5 had fewer notified cases than expected. In the analysis of areas within buffers per country (Table 3), there was a notable difference in the distribution of ASF notifications for each ecoregion. Cluster 6 contained more than half of the total ASF reports, and for the majority of countries (with the exception of the Czech Republic), the observed cases exceeded the expected number of notifications within the 0.045 decimal degrees (approximately 5 km) buffer (Table 3). The next ecoregion in terms of more notifications is number 1, with 28% of the cases. In this case, this is the largest ecoregion in the entire area of study; however, the number of expected cases was greater than the actual number of cases (Tables 2 and 3). Within the EU ASF-affected countries, only the surrounding areas of notifications of Italy had less than 13% coverage of ecoregion 1 (S4 Figure). Ecoregion 3 had 11.43% of the notifications, which was a bit less than expected for some countries and moderately lower for others (Table 3). Ecoregion 5 was predominant in Estonia with 44.3% of its surface covered by it. Most of the cases located in this ecoregion correspond to Estonia and Latvia, with the former being greater than expected (Table 2). The rest of the ecoregions accumulated less than 2% of the ASF notifications in wild boar.

## 4. Discussion

In this study, we have defined nine ecoregions for the Western Palearctic realm based on their differences in terms of eco-climatic and host-related factors of ASF. This cartography tool will help to better understand the patterns of distribution and spread of ASF in wild boars for wildlife management and other epidemiological studies in Europe. Furthermore, the tool proposed here may also prove useful against other infectious diseases transmitted by wild boar and to improve efforts at surveillance and control of wild boar diseases in the field.

Other studies defined bioclimatic regions or ecoregions [15, 50], and others performed a similar methodological approach as the one presented here to define bioclimatic regions for wild boar [51, 52]. In those last studies, the authors presented four homogeneous regions (Asian or northern, eastern, western, and southern), without any heterogenic intragroups. Although their northern area corresponds to our cold areas (ecoregions 4, 5, and 8), the ecoregions we defined for the ASF do not coincide with previous work developed for wild boar distribution [51, 52]. Furthermore, other studies developed a similar tool with climatic and environmental variables related to vector-borne disease at a lower geographical scale. This tool was conceived as a generic tool for vector-borne diseases; thus, the host of the pathogen was not included [13]. Unlike those studies, we defined the ecoregions for the current most important disease in swine due to its sanitary and economic consequences: ASF [53]. Our study contains not only the probability of the presence of wild boars but also their habitat suitability [16, 27]. This allows a more specific tool that was developed for the ASF context but has the potential applicability for other diseases with a strong relationship between domestic and wild suids, such as classical swine fever or Aujeszky's disease [10, 54].

On the other hand, this tool focused mainly on Western Palearctic wild boar excluding domestic pigs since we did not include the distribution of domestic pig farms or animal density. The stewardship of the disease is more challenging in wild populations than in livestock holdings, where interventions can be more easily implemented. Proposed measures, namely, physical barriers, massive depopulation, or carcass removal, can be insufficient in certain situations and even considered unfeasible and economically challenging in certain scenarios [5]. For that reason, oral vaccination has become one of the most promising strategies in terms of disease control in wild boar, given the uncontrollable extension of the disease, but in combination with other measures [55, 56]. The definition of ecoregions at a large geographical scale with fine resolution (Western Palearctic realm) can help apply different vaccination strategies and attend to different wild boar distributions and ecological and sanitary conditions. Bait distribution in the field is essential for the effectiveness and efficiency of the vaccination strategy. In that sense, climatic and environmental factors, some of which were taken into account in the current study, can affect the organoleptic and functional properties of the vaccine baits and bait consumption by target species and determine the success of the vaccination program [10, 57]. Moreover, the ecoregions could mark differences in seasonal wild boar behavior and population dynamics due to meteorological and food availability [28].

The ecoregions defined here (Figure 1) can also help understand and study the natural and nonanthropogenic spread of the disease in wild populations, although it was not specifically studied here. It is worth noting that in some EU countries, such as Romania, Bulgaria, Slovakia, and Lithuania, there is a domestic pig cycle (with more than 5% of the notifications in domestic pigs [48]), which may be producing spillover to wild boar, so the wild boar cycle may not be the unique way of transmission in the latter population [5]. Some of the ecoregions depict areas where the landscape and climatic conditions are more favorable for wild boar abundance and movement (Figure 2). Thus, our tool can be combined with other procedures to estimate the speed of the spread of the disease using disease spread models [58, 59] and to predict how it could potentially spread based on landscape connectivity [60]. For now, we can infer that areas where the disease has not yet arrived but have favorable ecoregions for ASF presence, are susceptible to concentrating a higher number of outbreaks there, in case there is an introduction of the disease there in comparison with less favorable ecoregions in the area. This information can help authorities from free areas to design management and risk-based surveillance measures, also in combination with other tools. The early detection of ASF, since neither commercialized vaccine nor treatment is available, has been proven crucial to its eradication in countries where a single incursion affecting the wild boar population in one defined area occurred [61]. Such was the case in Belgium and the Czech Republic, which implemented good early detection and control measures for eradicating the disease [62, 63]. Eradicating or minimizing the spread of the disease can be greatly beneficial due to the devastating sanitary and socioeconomic consequences of an ASF incursion [4, 53]. In that sense, providing ecoregions where it is more likely to find ASF in wild boar could improve the early detection of the disease in free areas through the intensification of surveillance efforts in those ecoregions.

In the analysis of ecoregions per country and ASF notifications, ecoregion 6, located in north-central Europe, had significantly more ASF notifications than the rest, with more than 51.5%, as well as a consistent and overall high presence in surrounding areas of notifications (Table 3, S4 Figure), showing its importance in the presence of the disease for the actual epidemic situation in Europe. Despite ASF cases not being notified more than expected in the Czech Republic, notifications were clustered in a very small area, and in the surroundings, ecoregion 6 was predominant. Furthermore, ecoregion 3, located mainly in southern Europe and containing around 11% of the ASF notifications, is not well represented in the overall analysis because it is more prevalent in Mediterranean regions, where ASF has not yet spread (Figure 1). However, in Italy, most cases fall into ecoregion 3, although ecoregion 6 contains more notifications than expected based on its extent (Table 3). It is worth noting that the Iberian Peninsula was affected by ASF from 1960 until 1995. Ecoregion 3 is predominant in this area and also in the provinces where the majority of outbreaks in low-biosecurity domestic pig holdings were notified [49]. In Spain, *Ornithodoros erraticus* ticks played a key role in ASF maintenance in this now-identified ecoregion 3 [64]. According to a study that evaluated the *Ornithodoros* genus suitability [65], the Mediterranean region, represented here mainly by ecoregion 3, was identified as highly suitable for *Ornithodoros* ticks. Therefore, there might be an association between mainland ecoregion 3 and the possible distribution of ticks that needs to be further investigated.

Ecoregion 1, located in north-central Europe, contained around 28% of the ASF notifications, significantly below the expected amount due to the extension of the region (Table 2). However, these herbaceous areas are highly represented in the vicinity of most EU ASF-infected zones (S4 Figure). Thus, this is a relevant ecoregion for ASF in wild boar in comparison with the majority of other ecoregions, but it might not be as important as ecoregions 6 and 3 (Table 2). Ecoregion 5 gathered 7.35% of the cases, mainly in Estonia and Latvia. Although the probability of wild boar presence is below the mean, the quality of habitat, along with its land cover characteristics, explains the suitability of ASF cases in wild boar in association with favorable ecoregions (Figure 2, S4 Figure). Ecoregions 2, 4, 7, 8, and 9 contain less than 2% of all ASF notifications in total. Ecoregions belonging to the northwestern Palearctic realm correspond to tundra and subpolar areas with lower, and in some cases null, wild boar presence and, therefore, lower or null ASF presence [66]. Ecoregions 2 and 9 represent areas with no ASF presence in the Mediterranean Basin, and ecoregion 9 has lower wild boar suitability. Lastly, ecoregion 7 represents mountainous areas and contains the most important mountain ranges of the study area, mainly dispersed in central-south Europe, from the western Iberian mountain system to the east Anatolia and Caucasus mountains (Figure 1). This ecoregion is not represented as favorable in the analysis of ecoregions per country and ASF notifications. However, these results raise an issue with the probability of occurrence of wild boar in that ecoregion [27], which is one of the highest (Figure 2).

Bosch et al. analyzed the distribution of wild boar occurrences in Eurasia at different altitudes and found that most wild boar presences were located in areas with an altitude range from 0 meters to 1,200 meters above sea level. Only a marginal presence of wild boar in Eurasia was reported at altitudes below 0 meters and above 2,400 meters in particular periods of the year [28]. In a study conducted in Bulgaria, the authors compared the presence of wild boar with habitat suitability and found a high correlation between them [67]. Wild boar habitat quality suitability based on land cover was identified as an important factor associated with ASF disease in these studies [16, 67], as well as the changes in land use cover, as the most relevant factors in the risk of appearance and transmission of emerging infectious diseases at a global level [68, 69]. Given this evidence and the results we obtained in the current work, the wild boar presence may have been overestimated at higher altitudes in the prediction we used [27]. Some abundance models cover the extension of the study area; however, they are presented as categorical data [27], which is challenging to implement in our methodology, or are based exclusively on hunting data, with the corresponding bias that comes with different hunting pressure and data collection between countries [51]. To obtain more robust results in models, a combination of different tools (probability of presence, abundance, and QAH), as done here, can give more robust results, reducing and balancing the over- and subestimations for use in eco-epidemiological studies.

Some improvements could be considered for future studies. For instance, although a spatial resolution of 1 km^2^ is appropriate for the extension of the study and is dependent on the input variables [18, 27], a finer resolution could improve the analysis of bait placement in the field. We have not directly considered the role of ticks, although some of the environmental variables that were included are important for the arthropod's ecology [65]. On the other hand, we also have not considered scavenging activities which were considered not epidemiologically relevant and represent a minor risk for spreading ASF [70]. To improve the model, variables related to *Ornithodoros* spp. distribution and scavengers could be analyzed for their inclusion in the model.

## 5. Conclusion

To the best of our knowledge, this is the first attempt to classify the Western Palearctic realm territory in similar ecoregions according to variables associated with ecological and sanitary aspects of ASF distribution in wild boar. The spatial resolution of the model allows for decision-making at both large and small scales, making it a general and versatile tool. The epidemiological results obtained here could be used as a basis for other models to unravel the patterns of distribution and spread of the disease in wild populations in the European scenario. Moreover, it could serve as a tool to design and improve prevention, surveillance, and control strategies, and particularly oral vaccination programs for wild boar populations, considering changes in the seasonal activity patterns, movement, and dietary behavior of wild boars in different ecoregions at landscape level. This could ultimately help with the control and eradication of ASF. The applicability of this tool could be also extended to other diseases that affect wild boar, namely, Aujeszky's disease, classical swine fever, etc.

## Figures and Tables

**Table 1:** Contribution of variables to each principal component (PC).

Variables	Principal components (PCs)							
	PC1	PC2	PC3	PC4	PC5	PC6	PC7	PC8
Mean temperature	0.79	0.19	0.26	0.48	0.04	0.17	0.01	0.13
Land cover: bare	0.6	−0.24	−0.63	0.24	−0.18	−0.28	−0.11	0
Land cover: herbaceous	0.2	−0.54	0.74	−0.28	−0.07	0	−0.15	0.01
Land cover: trees	−0.57	0.73	−0.25	0.08	0.08	0.17	−0.17	0.01
Probability of presence of wild boar	0.45	0.68	0.21	−0.26	0.38	−0.29	0	0.01
QAH *∗* NDVI	−0.09	0.75	0.38	0	−0.52	−0.12	0.02	0
Altitude	0.51	0.12	−0.46	−0.68	−0.15	0.16	0.01	0.07
Mean sun radiation	0.94	0.24	0.05	0.05	0	0.19	−0.01	−0.15

**Table 2:** Complementary results for the χ^2^ goodness of fit test for the ASF notifications.

Cluster/Ecoregion	Observed cases (*n*)	Percentage of cases	Expected random cases^*∗*^	Residuals
1	9524	28.02	13926.02	−37.3
3	3887	11.43	3809.12	1.26
5	2500	7.35	3170.34	−11.91
6	17506	51.5	12511.52	44.65

^*∗*^ASF expected cases if they were randomly distributed based on the size of the ecoregions. *χ*^2^ = 3528.6, *df* = 3, and *p* value <2.2^−16^.

**Table 3:** Comparison of the difference between the observed ASF notifications by country and the number of expected cases calculated from the expected probability inside the 0.045 decimal degree buffer, which is based on the number of cells per each ecoregion.

Ecoregions		Belgium	Bulgaria	Czech Republic	Estonia	Germany	Hungary	Italy	Latvia	Lithuania	Poland	Romania	Slovakia
1	Expected probability	13.34	17.62	24.52	20.59	47.48	56.38	10.24	24.86	46.2	58.45	44.44	34.77
	Percentage of cases	0.9	11.89	25.74	16.97	38.33	21.3	2.42	19.29	22.58	41.93	28.77	30.73
	Observed cases	4	87	96	444	358	1042	19	911	815	4636	977	134

2	Expected probability	0	3.96	0	0	1.04	0.57	2.5	0	0	0.02	4.24	0.19
	Percentage of cases	0	4.37	0	0	2.89	0.02	1.15	0	0	0	3.3	0
	Observed cases	0	32	0	0	27	1	9	0	0	0	112	0

3	Expected probability	34.75	43.12	12.61	0.07	10.57	17.02	66.27	1.47	2.01	6.28	19.15	16
	Percentage of cases	11.04	46.31	12.06	0.11	11.03	20.73	60.2	1.52	3.19	7.8	21.53	18.81
	Observed cases	49	339	45	3	103	1014	472	72	115	862	731	82

4	Expected probability	0	0	0	1.92	0.27	0.01	0	0.8	1.01	0.52	0	0.07
	Percentage of cases	0	0	0	1.45	0.43	0	0	0.19	0.42	0.28	0	0
	Observed cases	0	0	0	38	4	0	0	9	15	31	0	0

5	Expected probability	0	0.07	0.73	38.7	3.07	0.6	0	14.91	8.26	3.85	0.35	0.45
	Percentage of cases	0	0	3.75	44.84	1.93	0.29	0	12.89	5.96	4	0.32	0.92
	Observed cases	0	0	14	1173	18	14	0	609	215	442	11	4

6	Expected probability	51.9	33.22	62.12	30	36.87	24.97	16.22	55.74	41.32	29.19	31.28	48.05
	Percentage of cases	88.06	36.07	58.45	35.78	44.86	57.6	35.71	65.32	67.25	44.28	45.85	49.54
	Observed cases	391	264	218	936	419	2818	280	3085	2427	4896	1557	216

7	Expected probability	0	1.26	0	0	0	0	0.92	0	0	0	0.2	0.09
	Percentage of cases	0	1.09	0	0	0	0	0.51	0	0	0	0.15	0
	Observed cases	0	8	0	0	0	0	4	0	0	0	5	0

8	Expected probability	0	0	0	0.61	0.62	0.06	0	0.93	0.95	1.21	0.02	0.15
	Percentage of cases	0	0	0	0.84	0.54	0.06	0	0.78	0.61	1.71	0.09	0
	Observed cases	0	0	0	22	5	3	0	37	22	189	3	0

9	Expected probability	0	0.03	0	0.01	0.01	0.01	0	0	0	0	0.03	0.02
	Percentage of cases	0	0.27	0	0	0	0	0	0	0	0	0	0
	Observed cases	0	2	0	0	0	0	0	0	0	0	0	0

## Data Availability

All variables used here are publicly available and their sources are detailed in the Materials and Methods section. The rest of the data are made available upon reasonable request to the corresponding author.

## Supplementary Materials

Supplementary Materials S1 Figure: Spatial representation of selected variables. Maps generated using ArcMap v10.8.1 (Esri®). S2 Figure: Pearson correlation matrix for the variables used in the principal component analysis. S3 Figure: Results of the gap statistics method for the selection of the optimal number of clusters. S4 Figure: Comparison of the (A) ecoregion composition of the EU ASF-affected countries in the study area with more than ten notifications in wild boar, and (B) ecoregion composition within the 0.045 decimal degrees radius buffer of those countries.
