# Supplementary material for: Eco-Sanitary Regionalization of Wild Boar (Sus scrofa) in the Western Palearctic Realm as a Tool for the Stewardship of African Swine Fever
Source: Transbound Emerg Dis. 2023 Mar 22;2023:8080496. doi: 10.1155/2023/8080496 (PMC12017046; doi:10.1155/2023/8080496)
Supplement: Supplementary Materials — S1 Figure: Spatial representation of selected variables. Maps generated using ArcMap v10.8.1 (Esri®). S2 Figure: Pearson correlation matrix for the variables used in the principal component analysis. S3 Figure: Results of the gap statistics method for the selection of the optimal number of clusters. S4 Figure: Comparison of the (A) ecoregion composition of the EU ASF-affected countries in the study area with more than ten notifications in wild boar, and (B) ecoregion composition within the 0.045 decimal degrees radius buffer of those countries. [file 8080496.f1.docx]

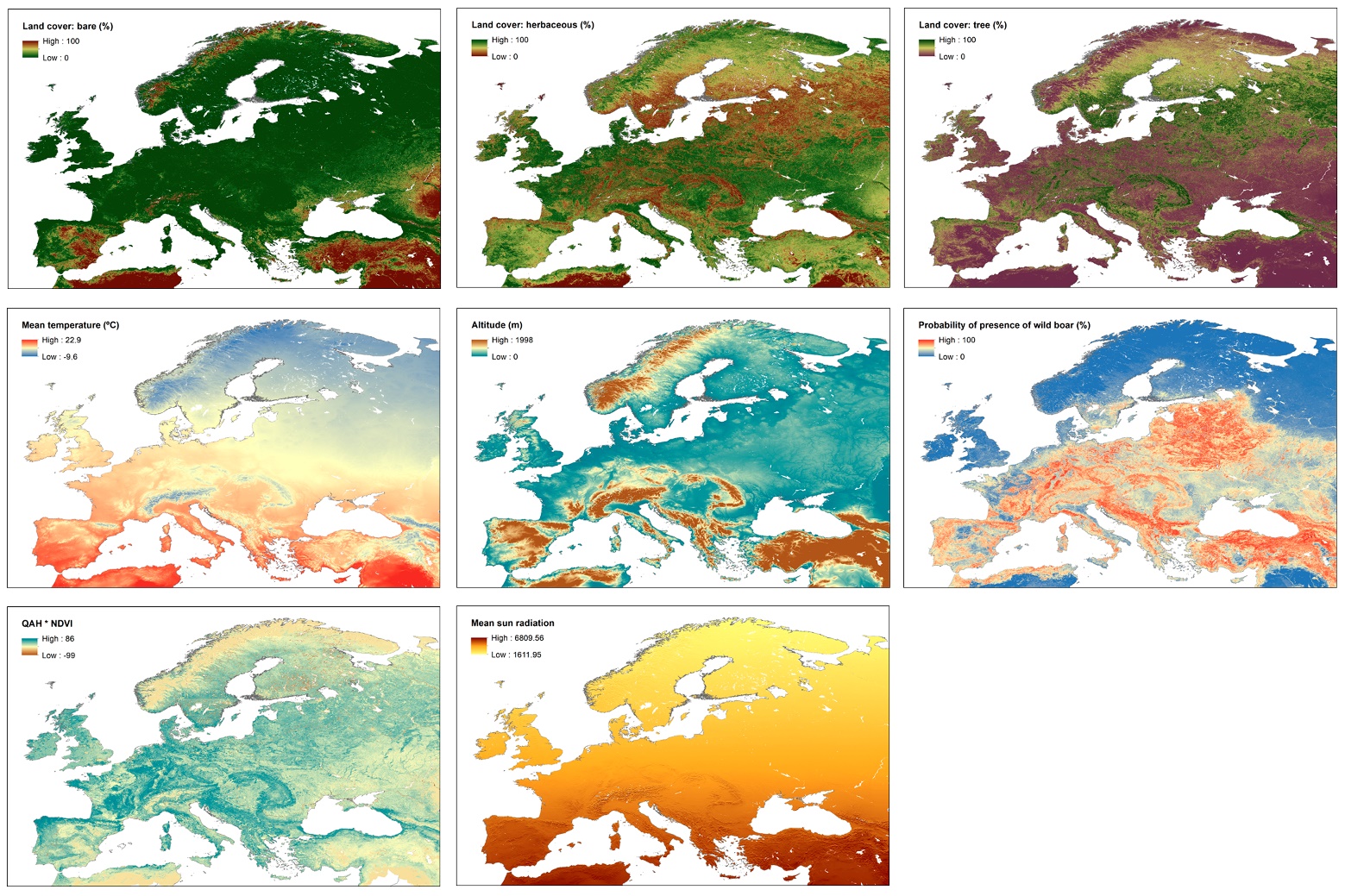


**S1 Figure.** Spatial representation of selected variables. Maps generated using ArcMap v10.8.1 (Esri^®^).


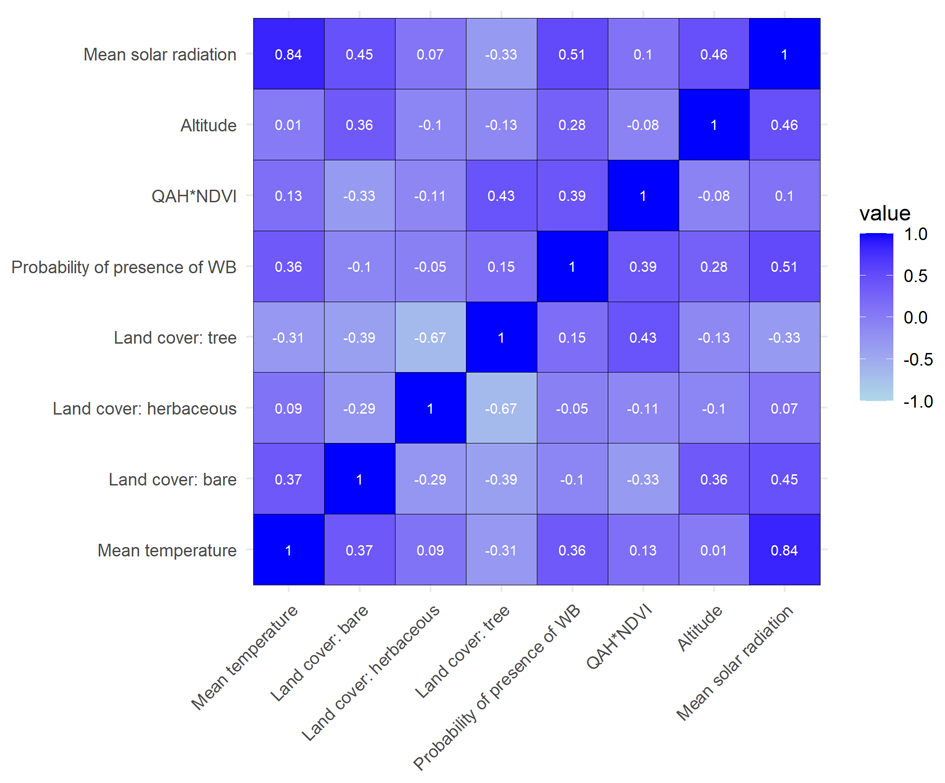


**S2 Figure.** Pearson correlation matrix for the variables used in the principal component analysis.


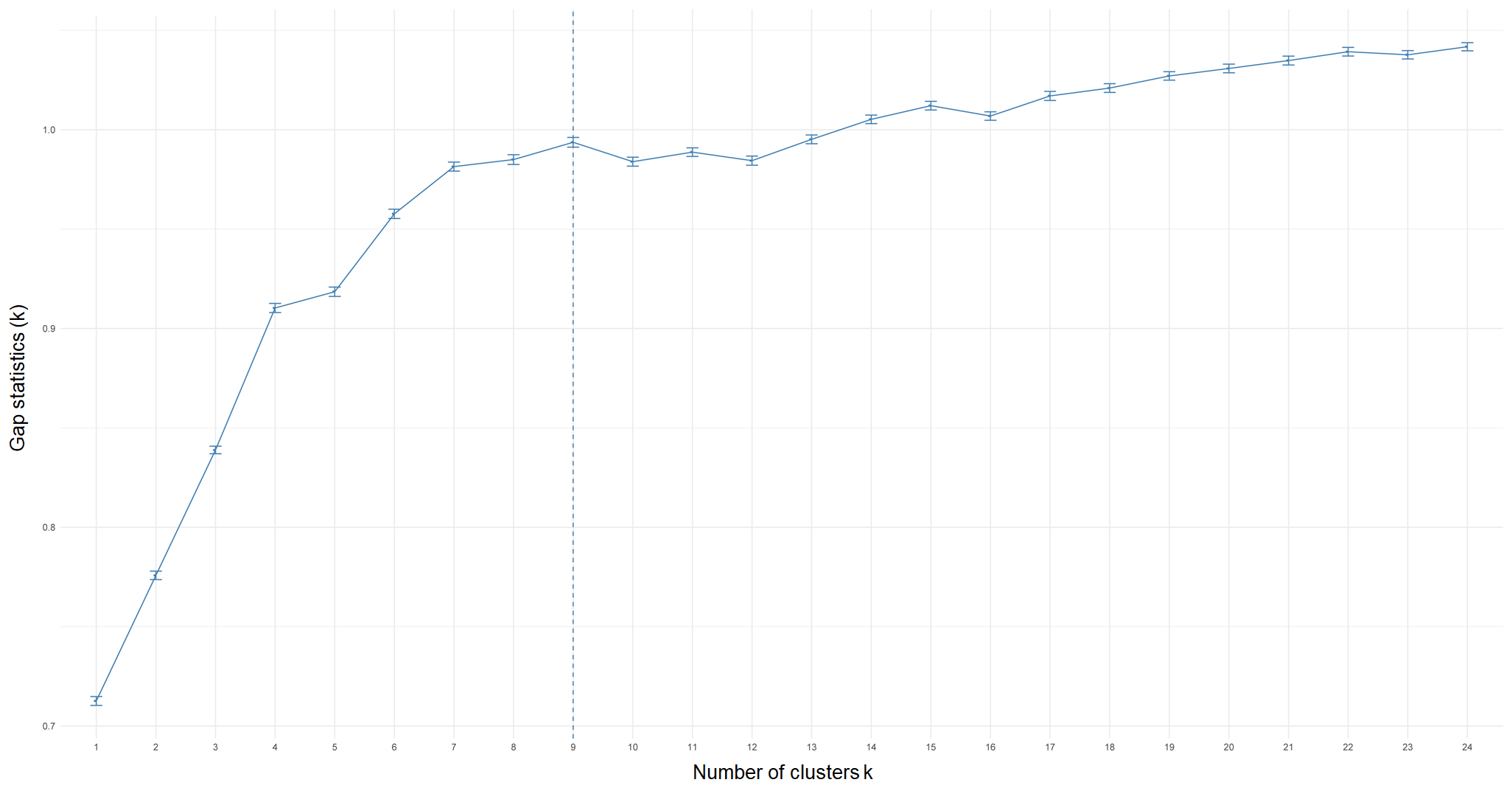


**S3 Figure.** Results of the gap statistics method for the selection of the optimal number of clusters.


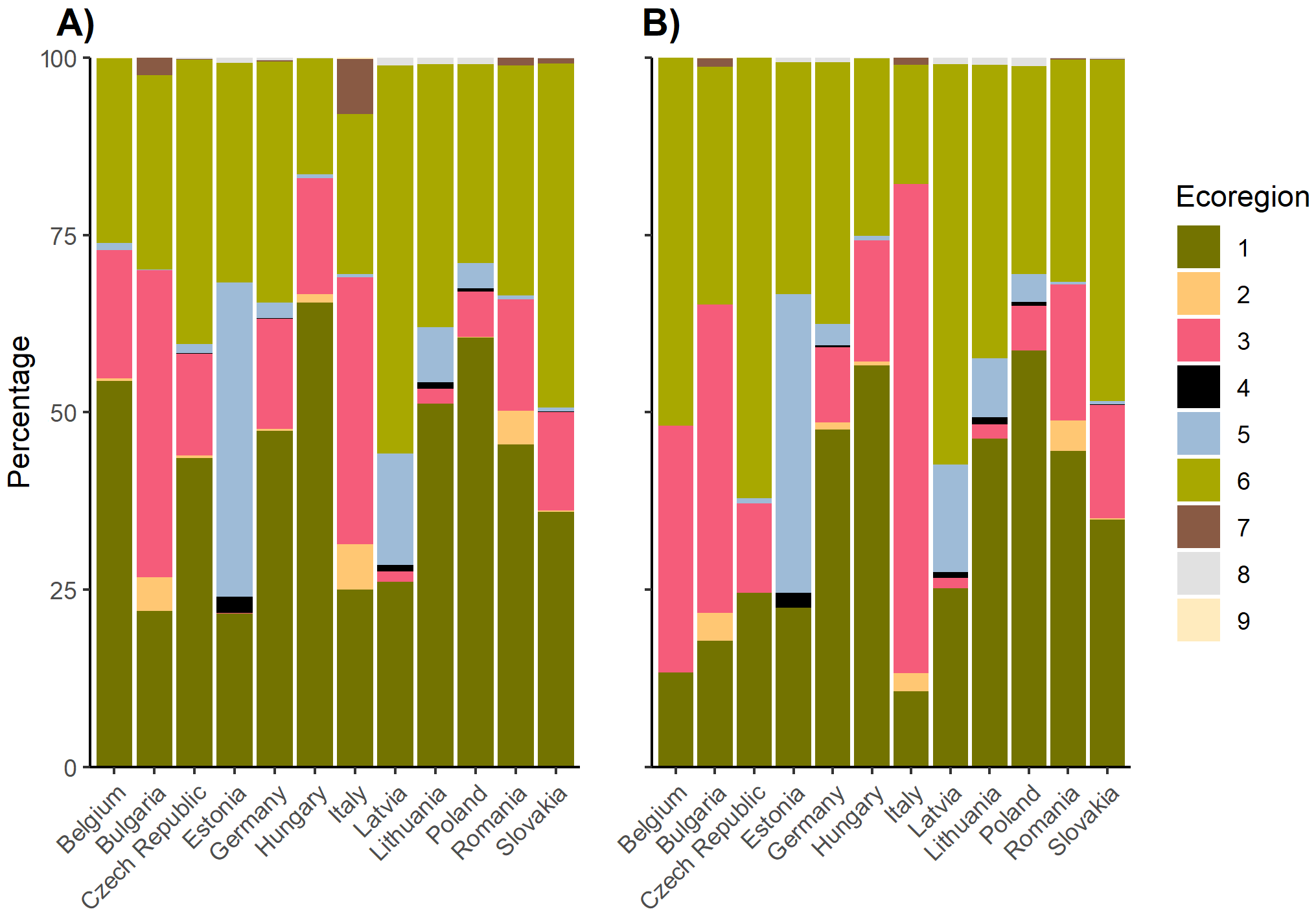


**S4 Figure.** Comparison of the (A) ecoregion composition of the EU ASF-affected countries in the study area with more than ten notifications in wild boar, and (B) ecoregion composition within the 0.045 decimal degrees radius buffer of those countries.
